# Artificial Intelligence (AI)-Enhanced Detection of Diabetic Retinopathy From Fundus Images: The Current Landscape and Future Directions

**DOI:** 10.7759/cureus.67844

**Published:** 2024-08-26

**Authors:** Lara Alsadoun, Husnain Ali, Muhammad Muaz Mushtaq, Maham Mushtaq, Mohammad Burhanuddin, Rahma Anwar, Maryyam Liaqat, Syed Faqeer Hussain Bokhari, Abdul Haseeb Hasan, Fazeel Ahmed

**Affiliations:** 1 Trauma and Orthopaedics, Chelsea and Westminster Hospital, London, GBR; 2 Medicine and Surgery, King Edward Medical University, Lahore, PAK; 3 Medicine, Bhaskar Medical College, Hyderabad, IND; 4 Surgery, King Edward Medical University, Lahore, PAK

**Keywords:** review, personalized medicine, screening, convolutional neural networks, fundus imaging, deep learning, artificial intelligence, diabetic retinopathy

## Abstract

Diabetic retinopathy (DR) remains a leading cause of vision loss worldwide, with early detection critical for preventing irreversible damage. This review explores the current landscape and future directions of artificial intelligence (AI)-enhanced detection of DR from fundus images. Recent advances in deep learning and computer vision have enabled AI systems to analyze retinal images with expert-level accuracy, potentially transforming DR screening. Key developments include convolutional neural networks achieving high sensitivity and specificity in detecting referable DR, multi-task learning approaches that can simultaneously detect and grade DR severity, and lightweight models enabling deployment on mobile devices. While these AI systems show promise in improving the efficiency and accessibility of DR screening, several challenges remain. These include ensuring generalizability across diverse populations, standardizing image acquisition and quality, addressing the "black box" nature of complex models, and integrating AI seamlessly into clinical workflows. Future directions in the field encompass explainable AI to enhance transparency, federated learning to leverage decentralized datasets, and the integration of AI with electronic health records and other diagnostic modalities. There is also growing potential for AI to contribute to personalized treatment planning and predictive analytics for disease progression. As the technology continues to evolve, maintaining a focus on rigorous clinical validation, ethical considerations, and real-world implementation will be crucial for realizing the full potential of AI-enhanced DR detection in improving global eye health outcomes.

## Introduction and background

Diabetic retinopathy (DR) is a microvascular complication of diabetes mellitus (DM) that affects the retina, potentially leading to vision impairment and blindness if left untreated. This condition progresses through several stages, beginning with mild nonproliferative diabetic retinopathy (NPDR), advancing to moderate and severe NPDR, and culminating in proliferative diabetic retinopathy (PDR). As the global prevalence of diabetes continues to rise, DR has emerged as a significant public health concern, affecting approximately one-third of individuals with diabetes worldwide [1]. The impact of DR on global health is substantial, with an estimated 103 million people affected by DR in 2020, a number projected to increase to 161 million by 2045 [1]. This growing burden underscores the critical need for effective screening and early detection strategies. Early identification of DR is crucial for preventing vision loss and reducing the socioeconomic impact of the disease. However, late-stage detection remains a significant challenge, often resulting in irreversible vision impairment and a diminished quality of life for affected individuals [2]. Current screening practices for DR typically involve regular dilated eye examinations performed by ophthalmologists or optometrists. These examinations include visual acuity tests, tonometry, and fundus photography or optical coherence tomography (OCT) to assess retinal changes [3]. While effective, these traditional screening methods face limitations, including the need for specialized equipment, trained personnel, and the time-intensive nature of the examinations. These factors contribute to suboptimal screening rates, particularly in resource-limited settings and areas with shortages of eye care professionals [4].

The advent of artificial intelligence (AI) in healthcare has opened new avenues for enhancing medical diagnostics and patient care. AI, particularly machine learning (ML) and deep learning (DL) algorithms, has demonstrated remarkable potential in analyzing complex medical data and images with high accuracy and efficiency [5]. In the field of ophthalmology, AI applications have shown promise in various areas, including the detection and classification of retinal diseases, prediction of disease progression, and personalized treatment planning [6]. The integration of AI in DR screening offers several potential advantages over traditional methods. AI-powered systems can analyze fundus images rapidly and accurately, potentially reducing the workload on healthcare professionals and improving screening efficiency [7]. Moreover, these systems can be deployed in primary care settings or even in remote areas through telemedicine platforms, potentially increasing access to screening services for underserved populations [8].

As research in this field progresses, AI algorithms are being developed to not only detect the presence of DR but also to grade its severity and identify specific lesions associated with the disease [9]. This capability could aid in more precise disease management and treatment planning. Additionally, AI systems have shown potential in predicting DR progression and identifying patients at high risk of developing vision-threatening complications, enabling proactive interventions [10]. However, the implementation of AI in DR screening is not without challenges. Issues such as algorithm interpretability, generalizability across diverse patient populations, integration with existing healthcare systems, and ethical considerations surrounding AI-assisted medical decision-making need to be carefully addressed [11]. As the field evolves, ongoing research aims to refine AI algorithms, validate their performance in real-world clinical settings, and develop best practices for their integration into ophthalmologic care.

This narrative review aims to explore the current state of AI-enhanced detection of DR from fundus images, examining the technologies involved, their potential benefits, and the challenges that need to be overcome for widespread clinical adoption. The study selection process involved comprehensive searches of databases like PubMed, Scopus, and Google Scholar, focusing on studies that evaluated the use of AI in DR screening. Inclusion criteria were studies that addressed AI technologies used for DR detection, classification, and progression prediction using fundoscopy images. Exclusion criteria included studies that lacked primary data, were published in non-peer-reviewed sources, or did not focus on AI applications in ophthalmology. The selected studies were critically appraised to ensure relevance and quality before being included in the review. By synthesizing the latest research and developments in this field, we seek to provide insights into how AI can revolutionize DR screening and contribute to improved eye health outcomes globally.

## Review

DR and fundus imaging

The pathophysiology of DR is complex and multifactorial, involving several biochemical pathways that are triggered by chronic hyperglycemia [12]. At the cellular level, prolonged hyperglycemia leads to increased oxidative stress, activation of protein kinase C, and accumulation of advanced glycation end-products (AGEs) [13]. These processes result in endothelial cell dysfunction, pericyte loss, and thickening of the basement membrane. As the disease progresses, these changes manifest as microaneurysms, the earliest clinically detectable sign of DR. At the vascular level, the loss of pericytes and endothelial cells leads to increased vascular permeability and the formation of acellular capillaries. This results in areas of retinal non-perfusion, which triggers the release of vascular endothelial growth factor (VEGF) and other angiogenic factors. Consequently, neovascularization occurs, characterizing the proliferative stage of DR [14,15].

Fundus photography is the cornerstone of DR screening and diagnosis [16]. This non-invasive imaging technique captures detailed images of the retina, optic disc, and macula using a specialized camera. Other imaging modalities used in DR assessment include fluorescein angiography, which helps visualize retinal blood flow and vascular leakage, and OCT, which provides cross-sectional images of the retina [17]. Traditionally, the detection and grading of DR have relied on manual evaluation of fundus images by trained ophthalmologists or retinal specialists. This process involves identifying and classifying various DR lesions, such as microaneurysms, hemorrhages, hard exudates, and neovascularization. However, manual grading faces several challenges, including subjectivity, where inter-grader variability can lead to inconsistent diagnoses. Additionally, the time-consuming nature of manual evaluation makes it labor-intensive to process large numbers of images. There is also limited accessibility, with a shortage of trained specialists, particularly in underserved areas. These challenges have motivated the exploration of automated methods for DR detection and grading.

AI techniques for image analysis

AI refers to the development of computer systems capable of performing tasks that typically require human intelligence. ML, a subset of AI, involves algorithms that can learn from and make predictions or decisions based on data. In medical imaging, common types of AI/ML approaches include supervised learning, where algorithms are trained on labeled datasets to classify images or detect specific features. Unsupervised learning involves algorithms identifying patterns in data without predefined labels. DL, a subset of ML, employs artificial neural networks (ANNs) with multiple layers to learn hierarchical representations of data. Early efforts focused on automated detection of DR lesions using traditional image processing techniques. With the advent of DL in the 2010s, there has been a significant leap in the performance of AI systems for DR detection [18]. Landmark studies, such as those by Gulshan et al. in 2016 and Ting et al. in 2017, demonstrated the potential of DL algorithms to achieve expert-level performance in DR screening [19,20]. These advancements have paved the way for the development and implementation of AI-enhanced systems for DR detection, promising to address the challenges of traditional manual grading methods and improve the efficiency and accessibility of DR screening programs.

In the context of DR detection, ML algorithms can be trained on large datasets of fundus images to recognize patterns and features associated with the disease. DL utilizes ANNs with multiple layers to learn hierarchical representations of data. This approach has shown remarkable success in analyzing complex medical images, including retinal fundus photographs. Neural networks, inspired by the human brain's structure, consist of interconnected nodes (neurons) that process and transmit information. In medical image analysis, neural networks can learn to identify intricate patterns and subtle changes in retinal images that may indicate DR [21-23].

Convolutional neural networks (CNNs) have emerged as a powerful DL architecture for image analysis, particularly in the medical field. The architecture of CNNs is specifically designed to process grid-like data, such as images, making them highly suitable for analyzing fundus photographs. CNNs consist of multiple layers, including convolutional layers, pooling layers, and fully connected layers. The convolutional layers apply filters to input images, extracting relevant features at different scales. Pooling layers reduce the spatial dimensions of the feature maps, while fully connected layers combine these features for final classification or prediction [24]. CNNs are particularly well-suited for image analysis due to their ability to automatically learn hierarchical features from raw image data. This characteristic allows CNNs to capture both low-level features (e.g., edges and textures) and high-level abstract representations (e.g., lesions or vessel abnormalities) in retinal images, making them highly effective in detecting DR [25].

Transfer learning is an ML technique that leverages knowledge gained from one task to improve performance on a different, but related, task. In the context of medical imaging, transfer learning involves using pre-trained models on large datasets of natural images and fine-tuning them for specific medical image analysis tasks [26]. The concept of transfer learning is particularly advantageous in medical image analysis for several reasons. Firstly, it addresses the common challenge of limited labeled medical image data by allowing models to benefit from the feature extraction capabilities learned from vast datasets of natural images. Secondly, transfer learning can significantly reduce the time and computational resources required for training complex DL models from scratch. By starting with pre-trained weights, models can converge faster and achieve better performance with less training data [27]. In DR detection, transfer learning has been successfully applied to adapt popular CNN architectures pre-trained on ImageNet (a large dataset of natural images) to analyze fundus images. This approach has shown promising results in improving the accuracy and efficiency of DR screening systems [23,28].

Development of AI models

The development of AI models for DR detection is a complex process that involves multiple stages, from data collection and preprocessing to model training, validation, and deployment. This process is crucial for creating accurate and reliable AI systems capable of assisting in the early detection and management of DR. The first step in developing AI models for DR detection is the collection of a large, diverse, and high-quality dataset of fundus images. These images should represent various stages of DR, from no retinopathy to proliferative DR, and include a wide range of patient demographics to ensure the generalizability of the model. The images are typically sourced from clinical databases, research institutions, and public datasets such as EyePACS or the MESSIDOR database. It is essential to have these images accurately labeled by experienced ophthalmologists to provide ground truth for training and evaluation.

Once the dataset is collected, preprocessing is a critical step to enhance the quality and consistency of the images. This may involve techniques such as contrast enhancement, noise reduction, and normalization to account for variations in image quality and acquisition conditions [29]. Additionally, data augmentation techniques, such as rotation, flipping, and color jittering, are often employed to artificially expand the dataset and improve the model's robustness to variations in image characteristics [30]. The choice of AI architecture is a crucial decision in model development. While various ML algorithms have been explored for DR detection, DL approaches, particularly CNNs, have shown superior performance in recent years [31]. Popular CNN architectures such as Inception, ResNet, and DenseNet have been adapted and fine-tuned for DR detection tasks [32,33]. These models are typically pre-trained on large datasets of natural images (e.g., ImageNet) and then fine-tuned on the DR dataset, leveraging transfer learning to improve performance and reduce training time. The training process involves feeding the preprocessed images through the neural network and adjusting the parameters of model to minimize the difference between its predictions and the ground truth labels. This is typically done using optimization algorithms such as stochastic gradient descent or Adam [24]. During training, techniques like cross-validation are employed to assess the model's performance and prevent overfitting [9].

Model evaluation is a critical step to assess the AI performance and generalizability. Common metrics used in DR detection include sensitivity, specificity, accuracy, and area under the receiver operating characteristic curve (AUC-ROC). It is essential to evaluate the model on a separate test set that was not used during training to get an unbiased estimate of its performance. Additionally, external validation on datasets from different populations or healthcare settings is crucial to assess the model's robustness and generalizability [34]. Interpretability and explainability of AI models are becoming increasingly important in medical applications. Techniques such as gradient-weighted class activation mapping (Grad-CAM) or layerwise relevance propagation (LRP) can be used to visualize the regions of the fundus image that the model focuses on when making its predictions [35]. This not only helps in understanding the model's decision-making process but also builds trust among clinicians and patients.

As AI models for DR detection advance, there is a growing trend toward developing end-to-end systems that can not only detect the presence of DR but also grade its severity and identify specific lesions. These systems often employ multi-task learning approaches, where a single model is trained to perform multiple related tasks simultaneously, potentially improving overall performance and efficiency. The final stages of model development involve rigorous clinical validation and regulatory approval processes. This includes prospective studies to assess the model's performance in real-world clinical settings and comparison with human experts [36]. Regulatory bodies such as the FDA have developed frameworks for evaluating AI-based medical devices, and several AI systems for DR detection have already received regulatory clearance.

Key AI models and algorithms

The field of AI-enhanced detection of DR has seen significant advancements in recent years, with several key models and algorithms demonstrating promising results. These developments have been driven by the increasing availability of large, well-annotated datasets and the continuous improvement of DL architectures. One of the pioneering works in this domain was the DL system developed by Gulshan et al. in 2016 [19]. This model, based on the Inception-v3 architecture, was trained on a dataset of 128,175 retinal images and achieved high sensitivity and specificity for detecting referable DR. The study demonstrated that DL algorithms could perform on par with board-certified ophthalmologists, setting a new benchmark for automated DR detection. Building on this foundation, Ting et al. developed a DL system capable of detecting multiple eye diseases, including DR, from retinal photographs. Their model, trained on 494,661 images, showed high sensitivity and specificity for referable DR detection. Notably, this study demonstrated the potential for AI systems to screen for multiple retinal diseases simultaneously, enhancing the efficiency of eye screening programs [20].

The IDx-DR system, developed by Abramoff et al., marked a significant milestone as the first autonomous AI-based diagnostic system for DR detection to receive FDA approval [37]. This system uses a novel AI algorithm that combines DL with traditional computer vision techniques. In a pivotal clinical trial, IDx-DR demonstrated high sensitivity and specificity for detecting more than mild DR, paving the way for its implementation in clinical practice. The EyeArt AI screening system, developed by Eyenuk Inc., is another notable algorithm that has shown promising results in large-scale clinical trials [38]. This system uses a combination of DL algorithms to detect referable DR with high sensitivity and specificity. EyeArt's ability to provide real-time results and integrate with existing healthcare workflows has made it a valuable tool for DR screening programs. Researchers have also explored ensemble learning approaches, combining multiple AI models to improve overall performance. For instance, Gargeya and Leng developed an ensemble of deep CNNs that achieved high sensitivity and specificity for DR detection. Their approach demonstrated the potential of combining multiple models to enhance the robustness and accuracy of AI-based DR screening systems [39].

Recent advancements have focused on developing models that can not only detect DR but also identify specific lesions associated with the disease. For example, the work by Dai et al. introduced a DL system called DeepDR, which performs real-time image quality assessment, lesion detection and segmentation, and DR grading. The system was trained on a large dataset of 466,247 fundus images from 121,342 patients with diabetes. DeepDR achieved high performance in detecting various retinal lesions, with AUCs of 0.901, 0.941, 0.954, and 0.967 for microaneurysms, cotton-wool spots, hard exudates, and hemorrhages, respectively. For DR grading, the system demonstrated excellent performance across all stages, with AUCs ranging from 0.943 to 0.972 for mild, moderate, severe, and proliferative DR. This multi-task approach provides detailed information to assisting clinicians in diagnosis and treatment planning, while also offering real-time feedback on image quality to improve screening efficiency [40]. The development of efficient and lightweight models for DR detection has also been an area of active research. Sait proposed a lightweight DL-based model for DR severity grading, designed to operate efficiently with limited computational resources. The model leverages the MobileNet V3-Small architecture, enhanced by feature extraction via YOLOv7 and feature selection using a modified quantum marine predator algorithm (QMPA). Key aspects of this model include image pre-processing with CLAHE and Wiener filter techniques to improve image quality, feature extraction with YOLOv7 to identify critical patterns related to DR severity, and feature selection using QMPA with a Cauchy-Gaussian mutation strategy. Classification is then performed using a hyperparameter-optimized MobileNet V3-Small model. The model demonstrated high accuracy (98.0% and 98.4%) and F1 scores (93.7% and 93.1%) on the APTOS and EyePACS datasets, respectively. Notably, it required fewer parameters, fewer floating-point operations (FLOPs), a lower learning rate, and less training time compared to existing models. This efficient and accessible approach enables DR screening to be implemented on mobile devices or in remote locations with limited computational resources, meeting the need for practical DR detection solutions [41].

As the field progresses, there is a growing emphasis on developing AI models that can generalize well across diverse populations and imaging conditions. The work by Bellemo et al. demonstrated the potential of AI systems to maintain high performance when applied to different ethnic populations, addressing concerns about the generalizability of AI models in healthcare [42,43].

Challenges and limitations

The implementation of AI-enhanced detection of DR from fundus images, while promising, faces several significant challenges and limitations that need to be addressed for widespread clinical adoption and optimal patient care. One of the primary challenges is the issue of data quality and standardization. AI models require large, diverse, and high-quality datasets for training and validation. However, the variability in fundus image quality, acquisition protocols, and equipment across different healthcare settings can significantly impact model performance. Images may vary in terms of resolution, field of view, and color balance, potentially affecting the AI system's ability to detect subtle DR-related changes consistently. Efforts to establish standardized imaging protocols and quality control measures are essential to ensure the reliability and generalizability of AI models across different clinical environments [44].

The generalizability of AI models across diverse populations remains a critical concern. Most AI models for DR detection have been developed and validated using datasets from specific geographic regions or ethnic groups. However, the appearance of retinal features can vary significantly across different populations due to factors such as genetic diversity, prevalence of comorbidities, and environmental influences. A model trained predominantly on one population may not perform equally well when applied to another, potentially leading to biased or inaccurate results. This challenge underscores the need for inclusive datasets that represent a wide range of ethnicities, age groups, and comorbidities to develop truly robust and generalizable AI systems [6,21,45].

Interpretability and explainability of AI models pose another significant challenge in the clinical adoption of these technologies. Many advanced DL models operate as "black boxes," making it difficult for clinicians to understand the reasoning behind their predictions. This lack of transparency can lead to skepticism and reluctance to accept AI-assisted diagnoses. Developing methods to visualize and explain the decision-making process of AI models, such as attention maps or feature importance analyses, is crucial for building trust among healthcare professionals and facilitating the integration of AI into clinical workflows [46].

The regulatory landscape for AI-based medical devices is still evolving, presenting challenges for developers and healthcare providers. While some AI systems for DR detection have received regulatory approval, the rapid pace of AI development often outstrips the regulatory frameworks. Issues such as defining performance standards, ensuring patient safety, and addressing liability concerns in cases of AI-assisted misdiagnosis need careful consideration. Additionally, the need for continuous monitoring and updating of AI models to maintain performance over time poses unique regulatory challenges that are not typically encountered with traditional medical devices [47]. Integration of AI systems into existing healthcare workflows and infrastructure presents both technical and organizational challenges. Many healthcare systems rely on legacy software and hardware that may not be compatible with advanced AI technologies. Seamless integration of AI tools into electronic health records (EHRs), picture archiving and communication systems (PACS), and telemedicine platforms is essential for widespread adoption. Moreover, the implementation of AI systems may require changes in clinical workflows, necessitating staff training and potential resistance to change [48].

Ethical considerations surrounding the use of AI in healthcare also present significant challenges. Issues such as patient privacy, data ownership, and informed consent in the context of AI-assisted diagnosis need careful consideration. There are concerns about the potential for AI systems to perpetuate or exacerbate existing healthcare disparities if not implemented thoughtfully. Ensuring equitable access to AI-enhanced healthcare technologies across different socioeconomic groups and geographic regions is a critical ethical imperative. Lastly, the cost-effectiveness and economic impact of implementing AI systems for DR detection need to be carefully evaluated. While AI has the potential to improve screening efficiency and reduce healthcare costs in the long term, the initial investment in AI infrastructure, ongoing maintenance, and potential changes to reimbursement models present financial challenges for healthcare systems, particularly in resource-constrained settings.

Addressing these challenges requires a multidisciplinary approach involving collaboration between AI researchers, clinicians, policymakers, and ethicists. Ongoing research, rigorous clinical validation, and thoughtful implementation strategies are necessary to overcome these limitations and realize the full potential of AI-enhanced DR detection in improving eye care delivery worldwide.

Future directions and opportunities

The field of AI-enhanced detection of DR from fundus images is rapidly evolving, with emerging technologies and methodologies promising to revolutionize medical imaging. One of the most significant advancements is the development of explainable AI (XAI) models. Unlike traditional "black box" algorithms, XAI systems provide insights into their decision-making processes, enhancing trust and interpretability for healthcare professionals. This transparency is crucial for clinical adoption and regulatory approval. Another promising area is the application of federated learning in ophthalmology. This approach allows AI models to be trained on decentralized data sources without compromising patient privacy, addressing one of the primary concerns in healthcare AI. Federated learning could enable the development of more robust and generalizable models by leveraging diverse datasets from multiple institutions.

AI technologies hold immense potential for contributing to personalized treatment plans in DR management. By analyzing vast amounts of patient data, including genetic information, lifestyle factors, and treatment histories, AI algorithms can help identify patterns and predict individual patient responses to various interventions. This personalized approach could significantly improve treatment outcomes and patient quality of life. In the realm of predictive analytics, AI systems are being developed to forecast DR progression and identify patients at high risk of vision loss. A study by Arcadu et al. demonstrated the ability of DL models to predict the two-year progression of DR from baseline fundus images with high accuracy [49]. Such predictive capabilities could enable early intervention and more effective resource allocation in ophthalmology clinics.

While current AI applications in DR primarily focus on detection and classification, there is growing interest in expanding these technologies for prognosis and treatment planning. AI algorithms could potentially assist ophthalmologists in determining the most effective treatment strategies based on individual patient characteristics and disease progression patterns. Integration of AI-powered DR detection systems with EHRs and other diagnostic tools is another area of active research. This integration could provide a more comprehensive view of patient health, enabling better-informed clinical decision-making. For instance, combining AI analysis of fundus images with data from OCT scans and patient medical histories could offer a more nuanced understanding of DR progression and associated complications [50].

As these technologies continue to evolve, it is crucial to maintain a focus on clinical validation, ethical considerations, and real-world implementation challenges. Collaborative efforts between AI researchers, clinicians, and healthcare organizations will be essential to realize the full potential of AI in DR management.

## Conclusions

AI-enhanced detection of DR from fundus images represents a significant advancement in ophthalmic care, offering the potential to improve screening efficiency, accessibility, and early detection rates. As the field progresses, addressing challenges such as model interpretability, generalizability, and seamless clinical integration will be crucial. Future developments in XAI, federated learning, and integration with other diagnostic modalities promise to further enhance the capabilities and clinical utility of these systems. The evolution of AI in DR management extends beyond detection, with emerging applications in personalized treatment planning and predictive analytics. As we move forward, maintaining a balance between technological innovation and clinical validation will be essential. By fostering collaboration between AI researchers, clinicians, and healthcare organizations, we can work towards realizing the full potential of AI in improving eye health outcomes for patients with diabetes worldwide.
